# Simplification of a Three-Constant Intraocular Lens Calculation Formula to a Single-Constant Approach: The Haigis Formula

**DOI:** 10.3390/diagnostics16121938

**Published:** 2026-06-22

**Authors:** Achim Langenbucher, Nóra Szentmáry, Alan Cayless, Benjamin Fassbind, Iwan Bolzern, Peter Hoffmann, Jascha Armin Wendelstein

**Affiliations:** 1 Department of Experimental Ophthalmology, Saarland University, 66424 Homburg, Germany; nszentmary@gmail.com (N.S.); wendelsteinjascha@gmail.com (J.A.W.); 2 Department of Ophthalmology, Semmelweis University, 1085 Budapest, Hungary; 3 School of Physical Sciences, The Open University, Milton Keynes MK7 6AA, UK; alan.cayless@gmail.com; 4 Augen- und Laserklinik Castrop-Rauxel, 44575 Castrop-Rauxel, Germany; pupillenpeter@gmx.de; 5 Department of Ophthalmology, Ludwig-Maximilians-University, 80539 Munich, Germany

**Keywords:** intraocular lens, Haigis formula, cataract surgery, biometry, formula optimization

## Abstract

**Background/Objectives:** To derive and validate a simplified modification of the Haigis intraocular lens (IOL) power calculation formula by reducing the three-constant effective lens position (ELP) model to a single constant while introducing an optimized keratometer index and axial length correction. **Methods:** In this retrospective study, a large multicentric dataset (Dataset 1; 22,466 eyes, 113 IOL models) was used to optimize the Haigis constant triplet and keratometer index using nonlinear programming with Cooke’s axial length correction. A second independent dataset (Dataset 2; 3181 eyes, six IOL models) was used for cross-validation. Three approaches were compared: classical Haigis, modified triplet, and two single-constant models acting on IOL power (H1) or ELP (H2). **Results:** The optimized keratometer index (1.3296 ± 0.0003) was significantly lower than the classical value, indicating systematic overestimation of corneal power. Modified triplet and single-constant approaches achieved comparable or slightly lower prediction errors than the classical formula. The H1 approach showed marginally superior performance. Bootstrapping confirmed parameter stability. **Conclusions:** A single-constant modification of the Haigis formula incorporating an optimized keratometer index and axial length correction maintains prediction accuracy while simplifying clinical implementation.

## 1. Introduction

Most classical intraocular lens (IOL) power formulae are designed as vergence formulae based on paraxial Gaussian optics. For example, the third-generation formulae such as SRK/T [1,2], Hoffer Q [3,4], or Holladay 1 [5] use the corneal radius or keratometric power and axial length measures from biometry together with a formula constant (A constant, personalized ACD constant or Surgeon Factor respectively) to calculate the IOL power (IOLP) for any target refraction at the spectacle plane, or the predicted Refraction with a preset IOLP. However, the Haigis formula [6], one of the most popular classical formulae in Europe, uses a set of three formula constants, in addition to the corneal radius and axial length. These three constants form a linear regression model to define the effective lens position (ELP). The ELP is a fictitious construct, which is back-calculated from the biometric measures, the IOLP, and the postoperative refraction in order to match the biometry, IOLP, and postoperative spherical equivalent refraction in the vergence formula [7,8,9,10,11].

All classical vergence formulae, and probably many of the modern formulae [3,12,13], use a simplified pseudophakic model involving three refractive elements for the calculation: the target refraction, which describes the intended refraction after cataract surgery at the spectacle plane, the cornea, which is simplified to a “thin lens” model [14,15] where the corneal front surface radius is converted to corneal power using a keratometer index (nK), and an IOL, which is again simplified to a “thin lens” model characterized with a lens power IOLP instead of geometrical data and the refractive index of the IOL material.

With the classical IOL formulae, the main differences are the prediction model for the ELP [3,8] and the conversion of corneal radius as measured with the biometer to corneal power using an index nK [14,15]. All classical formulae use the formula constants together with some selected biometric measures to systematically shift the lens (with the ELP) anteriorly or posteriorly, which means that formula constant optimization acts directly on the axial IOL position [3,7,12,16].

Whereas the SRK/T [1,2], Hoffer Q [4], and Holladay 1 [5] formula use advanced ELP models with hard and soft limitations, the Haigis formula [6] uses a multivariable linear regression setup in which the ELP is defined by ELP = a0 + a1∙ACD + a2∙AL where a0, a1, and a2 form the formula constant triplet and ACD and AL refer to the phakic anterior chamber depth and axial length measured with biometry before cataract surgery. In contrast to a single formula constant, the constant triplet gives some flexibility [9,10,11,17] to reduce or even eliminate trend errors for long or short eyes or eyes with a deep or flat anterior chamber. However, as the anterior chamber typically decreases over time with the (axial) growth of the crystalline lens, the Haigis formula bears the risk of an age dependency of the IOLP calculation where eyes from younger patients with a deeper ACD tend to end up in myopia and eyes of elderly patients with a more shallow ACD tend to end up in hyperopia [17,18]. Additionally, in contrast to optimizing a single formula constant, systematically larger datasets with sample sizes between *N* = 200 and *N* = 500 are required to appropriately optimize formula constant triplets, e.g., for the Haigis formula [8,9,10,11]. Online IOL constant optimization platforms such as IOLCon (https://IOLCon.org) and services described by Buonsanti et al. [19,20] address this challenge by pooling submissions from multiple clinical centers, thereby accumulating the large sample sizes required for reliable triplet optimization. The single-constant simplification proposed in the present study is directly complementary to this ecosystem: by reducing the optimization problem to a single free parameter, it enables reliable constant derivation from substantially smaller per-center datasets. For that purpose, simplified versions of formulae involving multiple constants (e.g., constant triplets) are highly recommended. At the same time, this would provide an opportunity to eliminate the overestimation of corneal power in the classical IOL formulae by using an excessive nK value.

The purpose of this paper is

• to derive a modification of the classical Haigis formula involving a single formula constant instead of a constant triplet and with a more realistic keratometer index avoiding systematic overestimation of corneal power,
• to investigate whether this formula constant should act as a shift in ELP such as in classical formulae or as a shift in IOLP, and
• to prove the clinical applicability and the performance of this modified Haigis formula in some clinical datasets containing preoperative biometry, power of the implanted IOL, and postoperative refraction.

## 2. Materials and Methods

### 2.1. Datasets for Our Study

Two datasets were used and analysed in this retrospective study:

The first dataset (Dataset 1) contains measurements from *N* = 22,466 eyes submitted to IOLCon (https://IOLCon.org) for lens constant optimization. This dataset includes preoperative biometric data (axial length AL, anterior chamber depth ACD measured from corneal front apex to the front apex of the crystalline lens, keratometric corneal front surface radius measured in the flat (R1 in mm) and steep meridians (R2 in mm) or keratometric power measured in the flat (K1 in dioptres (D)) and steep meridians (K2 in D) together with the keratometer index setting of the biometer (nK0), the power (IOLP in D), and type of the implanted IOL, and the postoperative refraction at the spectacle plane measured 4 to 12 weeks after cataract surgery (sphere REFS in D and Cylinder REFC in D). In total, data from 113 IOL types for capsular bag implantation from 21 IOL manufacturers were considered in Dataset 1. This dataset is used as reference in this study to derive the best-matching keratometer index (nKNF) and the formula constant triplet (a0NF, a1NF, and a2NF) for the new formula.

The second dataset (Dataset 2) contains *N* = 3181 clinical data from six modern IOL types on the market, which were recently uploaded to the IOLCon WEB platform for lens constant optimization. Dataset 2 is disjunct to Dataset 1. This dataset contains *N* = 142 data from the aspherical hydrophobic Alcon Clareon lens (Dataset 2A, Alcon, Fort Worth, TX, USA), *N* = 467 data from the aberration-neutral hydrophobic MX60 lens (Dataset 2B, Bausch & Lomb, Rochester, NY, USA), *N* = 821 data from the spherical hydrophobic SA60AT lens (Dataset 2C, Alcon, Fort Worth, USA), *N* = 254 data from the aspherical aberration-correcting hydrophobic SN60WF lens (Dataset 2D, Alcon, Fort Worth, USA), *N* = 885 data from the aspherical aberration-correcting hydrophobic Vivinex lens (Dataset 2E, Hoya Surgical, Singapore), and *N* = 612 data from the aspherical aberration-correcting Tecnis ZCB00 lens (Dataset 2F, Johnson & Johnson Vision, Jacksonville, FL, USA).

Each subset of Dataset 2 (Datasets 2A to 2F) contained only one eye per individual, randomly selected in cases where data from both eyes were available. Eyes with a history of ocular surgery and eyes with any pathology affecting the refractive outcome after cataract surgery (ectatic corneal diseases, zonular weakness, uncontrolled ocular hypertension or glaucoma, and retinal pathologies) were discarded from the datasets at the clinical center prior to transfer. All eyes in the datasets showed a visual acuity of at least 0.2 logMAR at the postoperative follow-up examination to ensure a reliable postoperative refraction.

The study adhered to the tenets of the Declaration of Helsinki. All eyes in Dataset 2 underwent cataract surgery at the Augen- und Laserklinik Castrop-Rauxel, Castrop-Rauxel, Germany. The local Institutional Review Board (Ärztekammer des Saarlandes, registration number 157/21) provided a waiver for this study, and patient-informed consent was not required for this study. The data were transferred to us in an anonymised fashion, precluding back-tracing of the patient.

Dataset 2 contained preoperative biometric data from the IOLMaster 700 (Carl-Zeiss-Meditec, Jena, Germany), whereas Dataset 1 contained data from various biometers: mostly IOLMaster 700, but also Anterion (Heidelberg Engineering, Heidelberg, Germany), Pentacam AXL (Oculus, Wetzlar, Germany), LenStar900 (Haag-Streit, Köniz, Switzerland), and OA-2000 (Tomey, Nagoya, Japan).

### 2.2. Preprocessing of the Data

The anonymised Excel data (.xlsx-format) were imported into MATLAB (Matlab 2025b, MathWorks, Natick, MA, USA) for further processing with a custom data processing code. Where keratometric power was provided, we converted K1 and K2 from dioptric power to corneal front surface radii R1 and R2 using the keratometer setting nK from the biometer. For the corneal radius, we derived the harmonic mean of R1 and R2 as R = 2·R1·R2/(R1 + R2) [15,17]. The postoperative spherical equivalent refraction was derived as SEQ = REFS + ½·REFC. The cylinder was treated as a scalar quantity in this calculation, consistent with standard practice in IOL formula evaluation studies [3]. Vector decomposition of astigmatism was not applied, as the Haigis formula targets spherical equivalent refraction; residual cylinder introduces noise but not systematic bias, provided no systematic cylinder orientation is present in the dataset. We then implemented the sum-of-segments correction for the axial length measures as described by Cooke [21,22]: CMAL = 1.23854 + 0.95855·AL − 0.05467·LT. This correction accounts for the fact that optical biometers measure the optical path length through the eye, which requires assumptions about the group refractive indices of the ocular media. The conventional single-index approach systematically overestimates geometric AL in long eyes and underestimates it in short eyes because the relative contribution of the crystalline lens (with higher refractive index) varies with eye length. The sum-of-segments approach uses segmental path lengths (corneal thickness, ACD, lens thickness, vitreous depth) with their respective group refractive indices to obtain a more accurate geometric AL [21,22]. Biometers that already implement internal sum-of-segments correction (e.g., Alcon Argos) do not require external CMAL correction.

### 2.3. Modifications of the Classical Haigis Formula

In the first step, we used Dataset 1 to derive a best-match nKNF and a formula triplet that fits to an overall dataset containing data from various clinical centers and various modern IOL types. For that purpose, we parametrized the lens constant triplet and the keratometer index in the original Haigis formula and used the Cooke CMAL axial length correction. An iterative nonlinear sequential quadratic programming algorithm (SQP), as described in previous papers, was used to minimize the formula prediction error (PE, defined as the difference between the formula prediction and the achieved postoperative SEQ) as the target parameter by simultaneous variation of a0NF, a1NF, a2NF, and nKNF.

In a second (test and crossvalidation) step, we used the six subsets, Dataset 2A to Dataset 2F, from Dataset 2 to calculate the formula constants for the classical Haigis formula as a reference, and the formula constants for the following modifications of the formula:

(A) The a0/a1/a2 lens constant triplet with the classical Haigis formula (without CMAL correction) with a keratometer index of nK = 1.3315 as proposed by Haigis in 2000 as a reference to show the performance of the classical Haigis formula,
(B) The a0/a1/a2 lens constant triplet with the modified Haigis formula with a CMAL correction and a keratometer index of nKNF as derived from the reference based on Dataset 1,
(C) The H1 lens constant with the modified single constant version of the Haigis formula with preset a0NF/a1NF/a2NF, a preset keratometer index of nKNF, and a CMAL correction as derived from the reference based on Dataset 1, where the formula constant H1 shifts the IOLP (IOLP := IOLP + H1), and
(D) The H2 lens constant with the modified single constant version of the Haigis formula with preset a0NF/a1NF/a2NF, a preset keratometer index of nKNF, and a CMAL correction as derived from the reference based on Dataset 1 where the formula constant H2 shifts the ELP in a similar way to the a0 in the original Haigis formula (ELP := ELP + H2).

Constant optimization for the (sets of) constants (a0NF/a1NF/a2NF/nKNF with step 1 or a0/a1/a2, H1, or H2 in step 2) was performed by minimizing the root-mean-squared PE [8,9,10,11,23]. A step size tolerance of 1 × 10^−8^ and a function tolerance of 1 × 10^−10^ were used as the stopping criteria for the algorithm. The distributions of the predicted ELP and PE were calculated for the situation described in step 1 and for the four situations described in step 2 (A to D).

In the third step, to test the robustness of the formula constants and the keratometer index, we set up a bootstrapping strategy where all parameters in the dataset (biometric data, IOLP and SEQ) were sampled NB = 10,000 times with replacement. For each bootstrap sample, we calculated the optimized formula constants (a0NF/a1NF/a2NF/nKNF with step 1 or a0/a1/a2, H1, or H2 in step 2). From these NB = 10,000 bootstrapped formula constants [23] and keratometer indices we derived the arithmetic mean and the standard deviation, as well as the median and the lower and upper limits of the 95% confidence intervals.

### 2.4. Statistical Analysis and Data Presentation

Descriptive data are recorded in terms of their arithmetic mean, standard deviation (SD), median, and the lower and upper boundaries of the 95% confidence interval (2.5% and 97.5% quantiles). The distributions of the predicted ELP and the prediction error PE for the situation described in step 1 and the 4 situations described in step 2 are shown using cumulative distribution function plots (CDF).

## 3. Results

Table 1 summarizes the descriptive data for the relevant preoperative biometric measures together with the labelled lens power and the spherical equivalent postoperative refraction for Dataset 1 and for Dataset 2 containing data from six different intraocular lenses.

Table 2 displays the preset values and the results of the optimization process for the lens constants and the keratometer index (in bold letters). The preset values for formula modifications were extracted from Dataset 1, and the preset triplet lens constants a0NF/a1NF/a2NF yielded values of 0.8735/0.3843/0.1220. The preset value for the keratometer index was nKNF = 1.3296 compared to the slightly larger keratometer index used in the classical Haigis formula. A comparison between the classical Haigis formula (formula A) and the three modified formulae B, C, and D shows the main differences: with the keratometer index nK = 1.3315 and without the CMAL correction of the axial length, the ELP generated by A is systematically larger compared to the ELP generated by all three modifications B, C, and D. Overall, all formula modifications (B, with a lens constant triplet, as well as C and D with a single H1 or H2 constant) seem to show at least the same performance in terms of RMS PE, in most cases even slightly outperforming the classical Haigis formula. In a direct comparison of both single-lens constant modifications, formula modification C with H1 acting on the IOLP seems to be slightly superior to formula modification D with H2 acting on the ELP.

**Figure 1:**
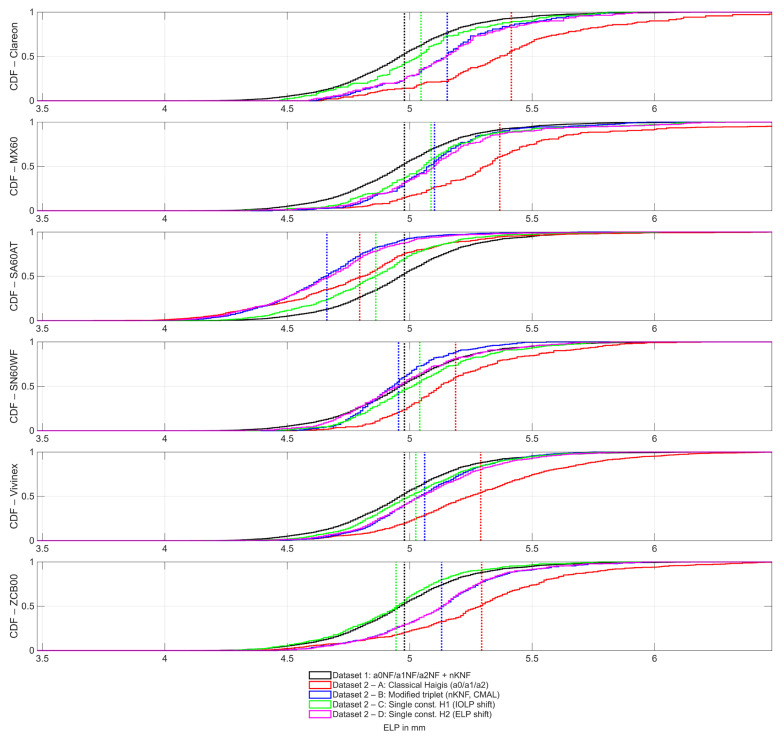
Cumulative distribution function (CDF) for the predicted effective lens position ELP: the black curves correspond to the CDF derived with Dataset 1 with constants a0NF/a1NF/a2NF and keratometer index nKNF, the red curves to the CDF with the classical Haigis formula (A, optimized constants a0/a1/a2 and nK = 1.3315), the blue curves to the CDF with the modified formula B (optimized constants a0/a1/a2 and nKNF), the green curves to CDF with the modified formula C with a single optimized lens constant H1 acting on the lens power IOLP (with keratometer index nKNF), and the magenta curves to CDF with the modified formula D with a single optimized lens constant H2 acting on the effective lens position ELP (similar to a0 in the classical Haigis formula, with keratometer index nKNF). The red, blue, green, and magenta curves were derived with Dataset 2 and show the situations with the Alcon Clareon lens (first graph), the Bausch & Lomb MX60 lens (second graph), the Alcon SA60AT lens (third graph), the Alcon SN60WF lens (fourth graph), the Hoya Vivinex lens (fifth graph), and the Johnson & Johnson Tecnis lens (sixth graph). The dotted vertical lines indicate the arithmetic mean of the ELP. We can see directly from the graph that the ELP with the classical Haigis formula is systematically larger compared to all formula modifications (apart from formula C for the SA60AT) due to the larger keratometer index of nK = 1.3315.

Figure 1 shows the CDF for the predicted ELP. With a constant triplet a0NF/a1NF/a2NF and keratometer index nKNF optimization based on the large Dataset 1, we see that the mean ELP is close to 5.0 mm, with nKNF = 1.3296. Due to the differences in the internal structure of the formula modifications, the ELP shows some variations between B, C, and D. For example, the ELP in modification D shows a constant offset H2 (horizontal shift in the CDF) as compared to modification C. This is because H2 adds directly to the ELP, while both formula modifications use the same preset values for a0NF/a1NF/a2NF. Comparing the ELP for the formula modifications B, C, and D with the ELP of the classical Haigis formula, we see that the classical Haigis formula produces the largest ELP with all of the IOLs, except the spherical SA60AT. With this lens model, the ELP with formula modification C slightly exceeds the ELP with the classical Haigis formula A. This is due to the large positive H1 constant (H1 = 0.41). If we consider formula modification D instead where the negative H2 constant (H2 = −0.18) adds to the ELP, the red CDF curve is again located right-hand-side of the magenta curve.

**Figure 2:**
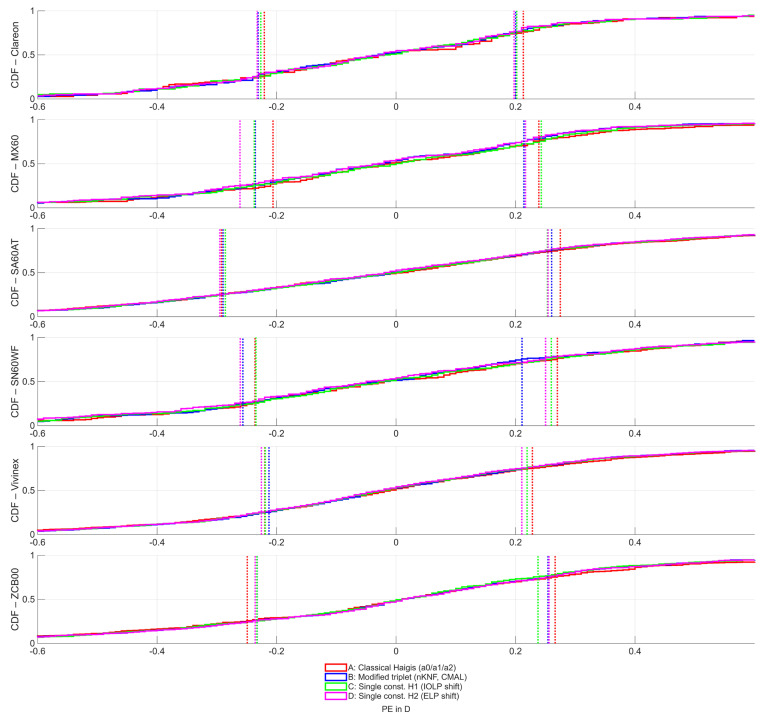
Cumulative distribution function (CDF) for the formula prediction error PE as a measure for the formula performance: the black curves corresponds to the CDF derived with Dataset 1 with constants a0NF/a1NF/a2NF and keratometer index nKNF, the red curves to the CDF with the classical Haigis formula (optimized constants a0/a1/a2 and nK = 1.3315), the blue curves to the CDF with the modified formula (optimized constants a0/a1/a2 and nKNF), the green curves to CDF with the modified formula with a single optimized lens constant H1 acting on the lens power IOLP (with keratometer index nKNF), and the magenta curves to CDF with the modified formula with a single optimized lens constant H2 acting on the effective lens position ELP (similar to a0 in the classical Haigis formula, with keratometer index nKNF). The red, blue, green, and magenta curves were derived with Dataset 2 (Dataset 2A to Dataset 2F) and show the situations with the Alcon Clareon lens (first graph), the Bausch & Lomb MX60 lens (second graph), the Alcon SA60AT lens (third graph), the Alcon SN60WF lens (fourth graph), the Hoya Vivinex lens (fifth graph), and the Johnson & Johnson Tecnis lens (sixth graph). The dotted vertical lines indicate the lower and upper boundaries of the interquartile ranges. We directly read out from the graph that the performance of all formula modifications (blue, green, and magenta) is comparable to the performance of the classical Haigis formula classical Haigis formula (red). Overall, the formula modification with nKNF with the triplet constant optimization (blue) seems to be slightly superior to the classical Haigis formula (red), and the modification with a single constant H1 acting at the lens power IOLP (green) seems to be slightly superior to the modification with a single constant H2 acting at the effective lens position ELP (magenta).

Figure 2 displays the performance curves of the classical Haigis formula and the formula modifications. The curves are quite similar for the classical Haigis formula A and the formula modifications B, C, and D. Comparing the various lens models under the test, we see that the PE for the Alcon Clareon, Hoya Vivinex, and Bausch & Lomb MX 60 lens are slightly smaller compared to the PE for the Alcon SA60AT or Johnson & Johnson Tecnis lens. The CDF curves also show that with some lens models, there is no exact mirror symmetry of the PE with respect to PE = 0. For example, for positive PE values, the classical Haigis formula A seems to have a slightly worse performance as compared to formula modifications B, C, and D, whereas for negative PE values, this is not systematically the case.

Table 3 shows the uncertainties of the optimized lens constants and of the optimized keratometer index derived with bootstrapping. The back-calculated optimized keratometer index nKNF shows a very small variation, with nKNF = 1.3296 ± 0.0003 and a 95% confidence interval from 1.3291 to 1.3302. This means that the keratometer index used in the classical Haigis formula (nK = 1.3315) is located outside the 95% bootstrapping confidence interval, and therefore systematically exceeds the back-calculated keratometer index and overestimates corneal power. From the standard deviation (and the 95% bootstrapping confidence interval), we learn that with a triplet constant optimization, a0 (SD up to 0.28) always shows a larger variation compared to a1 or a2 (SD up to 0.04). Both single constants (H1 and H2 for formula modifications C and D) show a variation up to SD = 0.03.

The proposed single-constant modification of the Haigis formula preserves the prediction performance of the original three-constant approach while substantially simplifying lens constant optimization. The incorporation of a revised keratometer index and Cooke axial length correction improves optical consistency and may facilitate broader clinical implementation in routine cataract surgery.

To provide the complete set of standard performance metrics recommended for IOL formula evaluation studies [3], Table A1 (Appendix A) lists the mean prediction error (ME), standard deviation (SD), median PE, mean absolute error (MAE), median absolute error (MedAE), interquartile range (IQR), and RMS PE for all four formula variants across the six IOL models of Dataset 2, together with the proportions of eyes with absolute PE within ±0.25, ±0.50, ±0.75, and ±1.00 D. Across all lens models and formula variants, ME was close to zero (range −0.029 to +0.015 D), confirming the absence of systematic bias after constant optimization. The RMS PE ranged from 0.347 D (formula B, Vivinex) to 0.416 D (formula A, SA60AT), with the proportion of eyes within ±0.50 D ranging from 76.5% to 88.0%. Formulae B, C, and D achieved comparable or slightly lower RMS PE compared with the classical Haigis formula A across all lens models, without any clinically meaningful loss in accuracy.

Table A2 and Table A3 (Appendix B) present subgroup analyses stratified by axial length (AL) and phakic anterior chamber depth (ACD), respectively. Across normal AL eyes (22–25 mm), all four formula variants showed well-balanced ME (range −0.019 to +0.027 D) and low RMS PE (0.335–0.393 D) for all lens models, confirming uniform performance in the central biometric range [24]. In short eyes (AL < 22 mm), formula A showed slightly higher ME in some lens subsets (e.g., +0.172 D for MX60), whereas formulae B, C, and D generally remained closer to zero. In long eyes (AL > 25 mm), some lens-specific trends were observed: for the MX60, formulae C and D showed a slightly more negative ME (−0.191 and −0.195 D, respectively) compared with formula A (+0.074 D), reflecting the influence of the preset triplet in eyes with larger axial dimensions. For the ACD subgroups, ME remained small and balanced across shallow, normal, and deep anterior chambers for all formula modifications, with no consistent directional trend attributable to either H1 or H2 simplification.

## 4. Discussion

Over the last 5 or 6 decades, many attempts have been made to improve lens power calculation with modifications of the historic Fyodorov or Gernet formulae [12,25,26,27,28,29,30,31,32,33,34,35,36]. More and more mostly non-disclosed formulae have been presented in the last 20 years, and some are still based on the simplistic pseudophakic eye model with three refractive surfaces, which was the fundament for the classical vergence formulae such as SRK/T [1,2], Hoffer Q [3,4], Holladay 1 [5], or the Haigis formula [6].

The fourth-generation Haigis formula used the lowest keratometer index of all the classical published formulae, but even with this keratometer index (nK = 1.3315), corneal power is, on average, overestimated, and we have to search for an appropriate keratometer index that does not overestimate corneal power during conversion from the measured corneal front surface radius [14,15,17].

In this paper, instead of directly calculating the keratometer index from any schematic model eye, e.g., by keeping the front-to-back curvature and corneal thickness constant, we back-calculated the keratometer index by simultaneously optimizing the lens constant triplet together with the keratometer index with a vergence formula according to Haigis using a nonlinear iterative optimization technique. Based on a large and clean multicentric dataset from IOLCon used for lens constant optimizations (Dataset 1, 113 lens models and 21 IOL manufacturers) with biometric data, the IOLP, and the refractive outcome, we found a keratometer index of nKNF = 1.3296, which is very close to the keratometer index of previous studies [14,15,37]. This value is lower than the classical Haigis value (1.3315) and lower than the Gullstrand thin-lens equivalent index, consistent with the fact that the posterior corneal surface exerts net negative power that is not captured by the anterior-surface thin-lens approximation. It is important to note that nKNF is applicable within the specific pipeline of this study: keratometric power from the biometer is first back-converted to anterior corneal radius using the biometer’s native index (nK0), and nKNF is then applied internally in the vergence formula. Applying nKNF directly to already-converted keratometric power values would produce incorrect results. In addition, to consider the systematic overestimation of the AL in long eyes and underestimation in short eyes, we decided to integrate the CMAL sum-of-segments setup of David Cooke [21,22], which is generally accepted in the literature. However, this AL correction is derived with data from the Haag-Streit LenStar, and a customization might be beneficial for other optical biometers [24]. With this nKNF and the CMAL correction, we extracted a lens constant triplet a0NF = 0.8735/a1NF = 0.3843/a2NF = 0.1222 as a preset value to be used in the single constant simplification of our vergence formula.

In the next step, we defined two versions of the modified formula involving a single-lens constant: in the first version (formula C), we use a lens constant H1, which does not interact with the ELP as in classical formulae, but instead adds to the IOLP. This concept refers to our philosophy that over the entire lens power range, an IOL design that is asymmetrically more curved at the front surface shifts its image-side principal plane (which refers to the power label of the lens according to the ISO standards) anteriorly, with the consequence that the lens (assuming positive IOLP) acts more strongly at the haptic plane, and a lens that is more curved at the back surface acts more weakly at the haptic plane. This offset between the refraction of the IOL in the haptic plane and the labelled IOLP (considered at the principal plane) is expressed as the H1 constant.

In the second version (formula D), we use a lens constant H2 that interacts with the ELP in a similar way to most classical formulae (e.g., the a0 constant in the Haigis formula). This concept refers to the philosophy that the design and assembly of the lens optics and haptics and the lens material may result in the IOL being located systematically more anteriorly (negative H2) or more posteriorly (positive H2) in the eye. However, even though this latter concept works quite well in normal or short eyes where an axial shift of the lens has a sufficient impact on the refraction, this concept does not work in very low-power lenses, and with negative-powered lenses, an anterior shift of the lens reduces the refractive power.

Both of the single-constant concepts (formulae C and D) may be directly compared in terms of the results shown in the tables and figures. To use the classical Haigis formula as a benchmark, we have also included this formula (formula A) with optimization of a constant triplet (using nK = 1.3315 without CMAL). Also, for a direct comparison between the upgraded formula using nKNF and CMAL to optimize the lens constant triplet a0/a1/a2 and the two formulae with a single lens constant (H1 or H2) with preset values for a0NF/a1NF/a2NF, we optimized the lens constant triplet a0/a1/a2 (using nKNF = 1.3296 with CMAL) in formula B.

To assist the reader with implementation of any consumer software (such as Microsoft Excel), we summarize the calculation steps as follows:

Axial length transformation CMAL = 1.23854 + 0.95855∙AL − 0.05467∙LT

With nK = 1.3296 and ELP0 = 0.8735 + 0.3843∙ACD + 0.1222∙AL

formula C with lens constant H1 acting to the IOLP reads

$$\mathrm{predicted}\;IOLP=-H1+\frac{1.336}{CMAL-ELP0}-\frac{1}{\frac{1}{\frac{TR}{1-0.012\cdot TR}+\frac{329.6}{R}}-\frac{ELP0}{1336}}$$

$$\mathrm{predicted}\;SEQ=\frac{1}{\frac{1}{\frac{1}{\frac{1}{\frac{1}{\frac{1336}{CMAL-ELP0}}-PIOL-H1}+\frac{CMAL-ELP0}{1336}}-\frac{329.6}{R}}+0.012}$$

and formula D with lens constant H2 acting to the ELP reads

$$\mathrm{predicted}\;IOLP=\frac{1.336}{CMAL-(ELP0+H2)}-\frac{1}{\frac{1}{\frac{TR}{1-0.012\cdot TR}+\frac{329.6}{R}}-\frac{\left(ELP0+H2\right)}{1336}}$$

$$\mathrm{predicted}\;SEQ=\frac{1}{\frac{1}{\frac{1}{\frac{1}{\frac{1}{\frac{1336}{CMAL-(ELP0+H2)}}-PIOL}+\frac{CMAL-(ELP0+H2)}{1336}}-\frac{329.6}{R}}+0.012}$$

where TR refers to the target refraction.

The most important findings of our study are (1) that with a customization of the Haigis formula (and by implication probably all classical formulae), the keratometer index used for internal conversion from corneal front surface radius to keratometric power [14,15] should be reduced to make the cornea model more realistic. (2) To avoid overestimation of axial length in long eyes (or underestimation in short eyes) with optical biometers that do not already implement an internal correction (such as the Alcon Argos device), a sum-of-segments correction [21,22] could help to ensure more reliable formula prediction results in long and short eyes. (3) With the modified keratometer index and implementation of CMAL, all lens constants (a0/a1/a2) have to be updated and newly optimized (please compare lens constant results of formula A and B). In clinical situations where lens constant triplets a0/a1/a2 are unavailable [7,8,27,28] or where not enough clinical results for a reliable constant triplet optimization are available [9,10,11], a simplification of the Haigis formula involving a single lens constant could be helpful [17]. By using appropriate preset constants (e.g., a0NF/a1NF/a2NF), together with nKNF and CMAL, we were able to show that the prediction performance of formula C with a lens constant H1 acting at the IOLP or formula D with a lens constant H2 are good alternatives to the triple constant version (formula B or the classical Haigis formula A), and the loss in prediction performance is with the datasets under test below clinical relevance. The specific practical advantages of the single-constant simplification are: (i) reliable H1 or H2 optimization requires approximately *N* = 100–200 eyes per lens model, compared with *N* = 200–500 for a reliable triplet optimization [8,9,10,11]; (ii) the single-constant approach avoids collinearity problems between a0 and a1 that can arise in small datasets; (iii) H1 is conceptually analogous to the A-constant familiar from SRK/T and related formulae, facilitating clinical adoption; (iv) the formula can be implemented in a standard spreadsheet without iterative optimization software; and (v) online platforms such as IOLCon can provide robust H1 or H2 estimates with smaller submission datasets, enabling a larger number of IOL models to be covered. In this context, it is worth relating our approach to the recent work of Buonsanti et al. [19,20], who describe online IOL calculation platforms and a novel constant optimization method based on zeroing the mean prediction error. Optimization to zero mean PE is a well-established mean-target approach that is efficient and performs well in practice. The concept of a multiplicative correction factor applied to IOL constants, as explored in that work, has conceptual overlap with the optimization factor framework previously described by Gatinel and evaluated clinically by our group [17]. The nonlinear SQP approach used in the present study differs in two important respects: it minimizes RMS PE rather than mean PE, which simultaneously reduces both systematic bias and random spread and is theoretically more robust in the presence of outliers; and it can simultaneously optimize multiple interdependent parameters (a0/a1/a2/nK) without requiring simplifying assumptions, which is not straightforwardly achievable with a univariate mean-zero strategy applied to a nonlinear multi-parameter vergence formula. The single-constant H1 or H2 formulation proposed here reduces to a univariate problem once the preset triplet is fixed, making it fully compatible with the mean-zero approach and with spreadsheet implementation for clinical use.

In a previous study [17], we investigated a concept for including an additional regression term in the classical Haigis formula to account for the central phakic lens thickness LT. With a back-calculated keratometer index of nK = 1.3294, which is very close to the keratometer index of the present study, and using an ELP prediction of ELP = a0 + a1∙ACD + a2∙AL + a3∙LT, we found that the performance of the Haigis formula could be significantly improved. Compared to a commercial non-disclosed lens power formula (K6 formula from David Cooke), this updated Haigis formula with four lens constants a0/a1/a2/a3 showed equivalent results tested with four clinical datasets [17]. However, we learned that in some clinical situations where there is no measurement data on the LT, the constant optimization with four lens constants might be challenging without applying nonlinear iterative optimization techniques, which is not available in many clinical centers. In other cases, there are simply not enough clinical data for lens constant optimization with four constants. For these reasons, we decided to work on a simplified version of a vergence formula with a single-lens constant.

However, our study has several limitations. First, the retrospective design and the registry-style nature of Dataset 1, which aggregates submissions from multiple centers, biometers, and IOL models, may introduce uncontrolled variability that cannot be fully accounted for in the analysis. Second, Dataset 2 originates from a single clinical center (Castrop-Rauxel) using a single biometer (IOLMaster 700), which limits the generalizability of the cross-validation results to other centers and biometers. Third, no prospective clinical validation has been performed; confirmation of the results in a prospective randomized study in a larger and more diverse population is required. Fourth, the CMAL axial length correction was derived from LenStar data [21,22] and may introduce residual biases for other optical biometers; device-specific customization of this correction would be beneficial. Fifth, subgroup sizes for short eyes (AL < 22 mm) were small in some lens subsets (*N* < 10 for Clareon and SN60WF), precluding reliable subgroup conclusions for these groups. Sixth, no direct comparison with modern non-disclosed formulas (Kane, EVO, Castrop) or online calculation platforms was performed, which limits benchmarking against the current state of the art. Seventh, to avoid complexity and to make this formula available for all optical biometers, we restricted the study to a thin-lens cornea model, which may be inappropriate after laser vision correction, with corneal edema, or with ectatic disease where the front-to-back curvature ratio is disturbed; in such cases, a vergence formula using a thick cornea model requiring corneal front and back surface data would be more appropriate. Eighth, we retained the core biometric inputs of the original Haigis formula (corneal radius, ACD, AL); including the central lens thickness might further enhance performance and reduce age-dependent bias. Ninth, only lens-constant interactions with IOLP or ELP were considered; other modifications such as interactions with the spectacle refraction plane were not investigated.

## 5. Conclusions

This paper presents a strategy of boosting the fourth-generation Haigis formula as a classical vergence-based lens power calculation formula by updating the keratometer index, using a sum-of-segments correction for the axial length measure, and by simplification from a lens constant triplet to a single constant, which could interact additively with the lens power or the effective lens position. By cross-validating with data from six modern intraocular lens models, we showed that these formula modifications exhibit at least the same performance as the classical Haigis formula, with the advantage that the handling and optimization of a single lens constant might be easier compared to a lens formula with a lens constant triplet. The clinical applicability should be confirmed with prospective randomized studies in a larger study population and with independent constant optimization.

## Figures and Tables

**Table 1:** Descriptive listing of the Dataset 1 derived from IOLCon to derive the best match keratometer index and constant triplet and Dataset 2 (subsets A to F) with six modern lenses in terms of the arithmetic mean, standard deviation (SD), median, and the lower (quantile 2.5%) and upper (quantile 97.5%) boundaries of the 95% confidence interval. Parameters listed are: axial length (AL), external phakic anterior chamber depth measured from the corneal front apex to the front apex of the crystalline lens (ACD), harmonic mean of the corneal front surface radius R, the power of the implanted lens (IOLP), and the postoperative spherical equivalent refraction (SEQ).

Explorative Description		AL in mm	ACD in mm	R in mm	IOLP in dpt	SEQ in dpt
Dataset 1: IOLCon data *N* = 22,466)	Mean	23.7949	3.1558	7.7214	21.2592	−0.5447
	SD	1.6259	0.4043	0.2637	4.4589	0.8331
	Median	23.6300	3.1700	7.7258	21.5000	−0.2500
	Quantile 2.5%	20.9000	2.3460	7.2229	10.5125	−2.7470
	Quantile 97.5%	27.5294	3.9440	8.2406	31.0000	0.5000
Dataset 2A: Alcon Clareon lens (*N* = 142)	Mean	24.2722	3.1833	7.7742	20.0986	−0.4296
	SD	1.5008	0.4301	0.2439	3.7304	0.9215
	Median	23.9450	3.2250	7.7871	21.0000	−0.1250
	Quantile 2.5%	22.0015	2.3400	7.3217	9.0500	−2.8687
	Quantile 97.5%	28.4345	3.9895	8.2347	25.4750	0.8687
Dataset 2B: B & L MX60 lens (*N* = 467)	Mean	24.4409	3.2443	7.7424	19.8330	−0.7586
	SD	2.0697	0.3521	0.2431	5.3032	0.8855
	Median	24.0408	3.2600	7.7290	21.0000	−0.5000
	Quantile 2.5%	21.4387	2.5859	7.3435	4.0000	−2.7500
	Quantile 97.5%	31.3795	3.9440	8.2075	29.0000	0.7500
Dataset 2C Alcon SA60AT lens (*N* = 821)	Mean	23.1467	3.0434	7.6977	22.7369	−0.4780
	SD	1.1507	0.3986	0.2656	4.5956	0.7152
	Median	23.1800	3.0260	7.7297	22.5000	−0.2500
	Quantile 2.5%	20.4510	2.3060	7.1052	13.5000	−2.6250
	Quantile 97.5%	26.4297	3.8180	8.1798	33.0000	0.5000
Dataset 2D Alcon SN60WF lens (*N* = 254)	Mean	24.2011	3.1920	7.6960	19.3563	−0.4358
	SD	1.4538	0.4042	0.2597	4.1085	0.7359
	Median	23.8700	3.1650	7.6942	20.0000	−0.2500
	Quantile 2.5%	21.8340	2.4185	7.2162	8.5000	−2.6437
	Quantile 97.5%	27.5345	4.0391	8.2408	25.5750	0.5000
Dataset 2E Hoya Vivinex lens (*N* = 885)	Mean	24.0932	3.1847	7.7643	20.6362	−0.5645
	SD	1.4039	0.4074	0.2682	3.7234	0.9230
	Median	23.9803	3.1845	7.7627	21.0000	−0.2500
	Quantile 2.5%	21.6745	2.3712	7.2685	12.0000	−2.5000
	Quantile 97.5%	27.3547	3.9441	8.3013	27.5000	0.5000
Dataset 2F J & J Tecnis lens (*N* = 612)	Mean	23.4607	3.1758	7.6734	22.3252	−0.5141
	SD	1.3917	0.4087	0.2634	3.9267	0.7902
	Median	23.3008	3.1908	7.6724	22.0000	−0.2500
	Quantile 2.5%	20.7967	2.3348	7.1747	14.0000	−2.5000
	Quantile 97.5%	26.9540	3.9820	8.2211	31.0000	0.5000

**Table 2:** Formula constants a0/a1/a2/a3 and keratometer index nK for the classical Haigis formula and the formula variations. The large Dataset 1 derived from IOLCon containing data from various lens models and clinical centers was used to derive the best match constant triplet a0NF/a1NF/a2NF and keratometer index nKNF (first row). In the lower part of the table, various formula modifications were tested with Dataset 2 (subsets A to F): ‘A’ refers to the classical Haigis formula with optimized lens constant triplets and a keratometer index 1.3315, ‘B’ refers to a modification with optimized lens constant triplets and nKNF for the keratometer index, ‘C’ refers to the modification with preset constant triplet a0NF/a1NF/a2NF and keratometer index nKNF with a single-lens constant H1 to be applied as an offset value for the lens power IOLP, and ‘D’ refers to the modification with preset constant triplet a0NF/a1NF/a2NF and keratometer index nKNF with a single lens constant H2 to be applied as an offset value for the effective lens position (ELP). The parameters optimized for the various models are marked in bold letters. The two rightmost columns list the back-calculated mean ELP and the root-mean-squared formula prediction error RMS PE. Sign convention for H1: a positive H1 value indicates that the labelled IOLP underestimates the effective refractive power at the haptic plane (the formula adds to IOLP); a negative H1 indicates overestimation (the formula subtracts from IOLP). Sign convention for H2: a positive H2 shifts the ELP posteriorly relative to the regression prediction; a negative H2 shifts it anteriorly. The variability of sign across IOL models reflects genuine differences in lens design geometry, not an inconsistency of the model.

Dataset	Formula	a0	a1	a2	H1	H2	nK	Mean ELP in mm	RMS PE in D
Dataset 1: IOLCon data (*N* = 22,466)	a0NF/a1NF/a2NF/nKNF	**0.8735**	**0.3843**	**0.1220**			**1.3296**	4.9947	0.4259
Dataset 2A: Alcon Clareon lens (*N* = 142)	A	**−0.7383**	**0.3602**	**0.2063**			1.3315	5.4148	0.3607
	B	**1.4397**	**0.3758**	**0.1046**			1.3296	5.1714	0.3487
	C	0.8735	0.3843	0.1220	**−0.2080**		1.3296	5.0611	0.3516
	D	0.8735	0.3843	0.1220		**0.1201**	1.3296	5.1811	0.3551
Dataset 2B: B & L MX60 lens (*N* = 467)	A	**−0.3779**	**0.3222**	**0.1923**			1.3315	5.3680	0.3874
	B	**2.1517**	**0.3551**	**0.0737**			1.3296	5.1023	0.3684
	C	0.8735	0.3843	0.1220	**−0.0355**		1.3296	5.1437	0.3748
	D	0.8735	0.3843	0.1220		**0.0394**	1.3296	5.1043	0.3823
Dataset 2C Alcon SA60AT lens (*N* = 821)	A	**0.7607**	**0.2925**	**0.2016**			1.3315	4.7958	0.4157
	B	**1.1770**	**0.3632**	**0.1033**			1.3239	4.6766	0.3997
	C	0.8735	0.3843	0.1220	**0.4050**		1.3296	4.8755	0.4011
	D	0.8735	0.3843	0.1220		**−0.1845**	1.3296	4.5911	0.4070
Dataset 2D Alcon SN60WF lens (*N* = 254)	A	**0.3624**	**0.2190**	**0.1705**			1.3315	5.1874	0.3728
	B	**2.2157**	**0.2888**	**0.0755**			1.3296	4.9635	0.3595
	C	0.8735	0.3843	0.1220	**0.1373**		1.3296	5.0566	0.3662
	D	0.8735	0.3843	0.1220		**−0.0590**	1.3296	4.9976	0.3770
Dataset 2E Hoya Vivinex lens (*N* = 885)	A	**−0.6979**	**0.3387**	**0.2030**			1.3315	5.2901	0.3682
	B	**1.4481**	**0.3739**	**0.1013**			1.3296	5.0784	0.3467
	C	0.8735	0.3843	0.1220	**−0.0837**		1.3296	5.0407	0.3483
	D	0.8735	0.3843	0.1220		**0.0484**	1.3296	5.0891	0.3557
Dataset 2F J & J Tecnis lens (*N* = 612)	A	**−1.1391**	**0.2908**	**0.2348**			1.3315	5.2940	0.4082
	B	**0.9171**	**0.3580**	**0.1317**			1.3296	5.1457	0.3811
	C	0.8735	0.3843	0.1220	**−0.3798**		1.3296	4.9631	0.3812
	D	0.8735	0.3843	0.1220		**0.1804**	1.3296	5.1434	0.3904

**Table 3:** Uncertainty of the formula constant triplets a0NF/a1NF/a2NF, a0/a1/a2, and single constants H1 and H2 using bootstrapping with NB = 10,000 bootstrap samples with replacement. The formula constants refer to the formula constants and the keratometer index marked in bold letters in Table 2. The data columns show the bootstrapped mean, standard deviation (SD), and the lower and upper boundaries of the 95% confidence intervals (2.5% and 97.5% quantiles). ‘A’ refers to the classical Haigis formula with optimized lens constant triplets and a keratometer index 1.3315, ‘B’ refers to a modification with optimized lens constant triplets and nKNF for the keratometer index, ‘C’ refers to the modification with preset constant triplet a0NF/a1NF/a2NF and keratometer index nKNF with a single lens constant H1 to be applied as an offset value for the lens power IOLP, and ‘D’ refers to the modification with preset constant triplet a0NF/a1NF/a2NF and keratometer index nKNF with a single lens constant H2 to be applied as an offset value for the effective lens position (ELP).

Dataset	Lens Constants and nK		Mean	SD	2.5% Quantile	97.5% Quantile
Dataset 1: IOLCon data (*N* = 22,466)	a0NF		0.8735	0.1000	0.6776	1.0695
	a1NF		0.3843	0.0030	0.3783	0.3902
	a2NF		0.1222	0.0048	0.1128	0.1317
	nKNF		1.3296	0.0003	1.3291	1.3302
Dataset 2A: Alcon Clareon lens (*N* = 142)	A	a0	−0.7383	0.2749	−1.2770	−0.1996
		a1	0.3602	0.0394	0.2829	0.0394
		a2	0.2063	0.0137	0.1795	0.0137
	B	a0	1.4397	0.2506	0.9485	1.9308
		a1	0.3759	0.0136	0.3492	0.0136
		a2	0.1046	0.0102	0.0846	0.0102
	C	H1	−0.2080	0.0301	−0.2671	−0.1489
	D	H2	0.1281	0.0158	0.0892	0.1509
Dataset 2B: B & L MX60 lens (*N* = 467)	A	a0	0.3779	0.2175	−0.8042	0.0485
		a1	0.3222	0.0306	0.2621	0.3822
		a2	0.1923	0.0104	0.1923	0.2127
	B	a0	2.1517	0.1404	1.8766	2.4268
		a1	0.3551	0.0080	0.3394	0.3707
		a2	0.0737	0.0055	0.0630	0.0844
	C	H1	−0.0355	0.0175	−0.0698	−0.0013
	D	H2	0.0394	0.0091	0.0215	0.0573
Dataset 2C Alcon SA60AT lens (*N* = 821)	A	a0	−0.7607	0.1273	−1.0102	−0.5112
		a1	0.2925	0.0214	0.2506	0.3344
		a2	0.2016	0.0065	0.1888	0.2143
	B	a0	1.1770	0.1231	0.9357	1.4183
		a1	0.3632	0.0144	0.3350	0.3913
		a2	0.1033	0.0057	0.0922	0.1145
	C	H1	0.4050	0.0140	0.3776	0.4324
	D	H2	−0.1845	0.0066	−0.1975	−0.1714
Dataset 2D Alcon SN60WF lens (*N* = 254)	A	a0	0.3624	0.2558	−0.1390	0.8638
		a1	0.2190	0.0344	0.1515	0.2865
		a2	0.1705	0.0116	0.1477	0.1933
	B	a0	2.2157	0.0194	2.1777	2.2537
		a1	0.2888	0.0302	0.2297	0.3479
		a2	0.0755	0.0041	0.0675	0.0835
	C	H1	0.1373	0.0231	0.0921	0.1825
	D	H2	−0.0590	0.0132	−0.0849	−0.0331
Dataset 2E Hoya Vivinex lens (*N* = 885)	A	a0	−0.6979	0.1258	−0.9445	−0.4513
		a1	0.3387	0.0189	0.3016	0.0189
		a2	0.2038	0.0061	0.1919	0.0061
	B	a0	1.4481	0.1135	1.2258	1.6705
		a1	0.3739	0.0031	0.3680	0.0030
		a2	0.1013	0.0044	0.0926	0.0044
	C	H1	−0.0837	0.0114	−0.1061	−0.0613
	D	H2	0.0484	0.0062	0.0363	0.0606
Dataset 2F J & J Tecnis lens (*N* = 612)	A	a0	−1.1391	0.1645	−1.4615	−0.8167
		a1	0.2908	0.0236	0.2445	0.3371
		a2	0.2348	0.0080	0.2192	0.2505
	B	a0	0.9171	0.1582	0.6071	1.2272
		a1	0.3580	0.0192	0.3205	0.3956
		a2	0.1317	0.0073	0.1174	0.1461
	C	H1	−0.3798	0.0131	−0.4095	−0.3502
	D	H2	0.1804	0.0075	0.1654	0.1953

## Data Availability

Data could be provided from the corresponding author upon serious request.

## Appendix A

**Table A1:** Standard performance metrics for Dataset 2. Prediction error metrics for the four formula variants across six IOL models in Dataset 2. Formula A: Classical Haigis (nK = 1.3315, no CMAL, arithmetic mean radius); B: Modified triplet (nKNF = 1.3296, CMAL, harmonic mean radius); C: Single-constant H1 (IOLP shift, preset a0NF/a1NF/a2NF, nKNF, CMAL); D: Single-constant H2 (ELP shift, preset a0NF/a1NF/a2NF, nKNF, CMAL). ME = mean prediction error; SD = standard deviation of PE; MedPE = median PE; MAE = mean absolute error; MedAE = median absolute error; IQR = interquartile range; RMS PE = root-mean-squared prediction error. All values in dioptres (D). Percentage columns give the proportion of eyes with |PE| within the stated threshold.

IOL (*N*)	Formula	ME (D)	SD (D)	MedPE (D)	MAE (D)	MedAE (D)	IQR (D)	RMS PE (D)	±0.25 D (%)	±0.50 D (%)	±0.75 D (%)	±1.00 D (%)
Clareon *N* = 142	A—Classical Haigis	0.006	0.362	−0.014	0.280	0.218	0.434	0.361	57.7	88.0	94.4	98.6
	B—Modified triplet	0.000	0.352	−0.024	0.270	0.220	0.431	0.351	62.0	88.0	94.4	98.6
	C—Single const. H1	0.001	0.357	−0.012	0.273	0.212	0.429	0.356	58.5	85.9	94.4	98.6
	D—Single const. H2	−0.005	0.353	−0.019	0.270	0.217	0.430	0.352	60.6	87.3	94.4	98.6
MX60 *N* = 467	A—Classical Haigis	0.015	0.388	−0.009	0.293	0.229	0.445	0.387	55.5	84.2	94.4	98.3
	B—Modified triplet	−0.018	0.371	−0.017	0.280	0.221	0.450	0.371	56.3	86.5	93.8	98.5
	C—Single const. H1	−0.003	0.383	0.009	0.291	0.240	0.481	0.383	52.2	86.1	94.4	97.6
	D—Single const. H2	−0.029	0.379	−0.024	0.288	0.231	0.478	0.380	55.2	84.8	92.9	97.9
SA60AT *N* = 821	A—Classical Haigis	0.005	0.416	0.019	0.334	0.281	0.567	0.416	44.8	76.5	92.6	98.5
	B—Modified triplet	0.002	0.400	0.002	0.320	0.278	0.550	0.400	46.5	79.4	93.9	98.9
	C—Single const. H1	0.000	0.403	0.004	0.323	0.269	0.539	0.402	46.2	78.7	94.0	98.9
	D—Single const. H2	−0.009	0.402	−0.010	0.322	0.267	0.549	0.402	46.5	78.6	94.5	98.8
SN60WF *N* = 254	A—Classical Haigis	0.007	0.374	−0.014	0.301	0.245	0.506	0.373	50.8	82.3	95.7	99.2
	B—Modified triplet	−0.012	0.363	−0.021	0.291	0.234	0.467	0.363	51.6	80.3	95.7	99.6
	C—Single const. H1	−0.002	0.376	−0.020	0.300	0.249	0.494	0.375	50.4	80.3	95.3	99.6
	D—Single const. H2	−0.027	0.380	−0.042	0.304	0.255	0.511	0.380	48.4	79.5	94.9	99.6
Vivinex *N* = 885	A—Classical Haigis	0.006	0.368	−0.005	0.282	0.223	0.448	0.368	54.5	83.2	95.0	98.6
	B—Modified triplet	0.002	0.348	−0.016	0.268	0.216	0.432	0.348	57.4	86.2	95.8	98.9
	C—Single const. H1	0.000	0.351	−0.016	0.269	0.219	0.438	0.351	55.9	85.8	95.6	98.8
	D—Single const. H2	−0.005	0.350	−0.019	0.269	0.220	0.436	0.350	56.6	85.8	95.9	98.8
ZCB00 *N* = 612	A—Classical Haigis	0.006	0.409	0.015	0.315	0.260	0.515	0.408	48.4	79.1	93.3	98.2
	B—Modified triplet	0.003	0.381	0.018	0.294	0.250	0.487	0.381	50.2	81.4	94.0	98.9
	C—Single const. H1	−0.001	0.381	0.012	0.292	0.236	0.471	0.381	52.8	81.9	93.6	98.9
	D—Single const. H2	0.005	0.381	0.018	0.294	0.247	0.492	0.381	50.5	81.2	94.1	99.0

## Appendix B

**Table A2:** Subgroup analysis by Axial Length. Mean prediction error (ME) and RMS PE stratified by axial length for each IOL model and formula variant. Short: AL < 22 mm; Normal: 22 ≤ AL ≤ 25 mm; Long: AL > 25 mm. Groups with fewer than 10 eyes are marked “—”. Formulas A–D as in Table A1. ME values prefixed with sign; positive = hyperopic shift.

IOL (*N*)	AL Subgroup (mm)	*n*	ME A (D)	ME B (D)	ME C (D)	ME D (D)	RMS A (D)	RMS B (D)	RMS C (D)	RMS D (D)
Clareon *N* = 142	Short (<22)	4	—	—	—	—	—	—	—	—
	Normal (22–25)	107	+0.003	+0.011	+0.032	+0.015	0.371	0.354	0.353	0.354
	Long (>25)	31	+0.023	−0.024	−0.105	−0.067	0.344	0.356	0.386	0.366
MX60 *N* = 467	Short (<22)	19	+0.172	+0.162	+0.374	+0.315	0.550	0.555	0.650	0.616
	Normal (22–25)	354	−0.009	+0.001	+0.027	−0.004	0.351	0.338	0.336	0.335
	Long (>25)	94	+0.074	−0.128	−0.191	−0.195	0.470	0.439	0.467	0.467
SA60AT *N* = 821	Short (<22)	164	+0.025	+0.010	−0.018	+0.075	0.484	0.466	0.470	0.467
	Normal (22–25)	588	−0.019	−0.009	−0.010	−0.034	0.393	0.379	0.381	0.384
	Long (>25)	69	+0.162	+0.075	+0.128	+0.007	0.429	0.400	0.406	0.389
SN60WF *N* = 254	Short (<22)	7	—	—	—	—	—	—	—	—
	Normal (22–25)	189	−0.001	+0.014	+0.036	+0.017	0.373	0.360	0.369	0.369
	Long (>25)	58	+0.028	−0.111	−0.157	−0.206	0.384	0.384	0.397	0.419
Vivinex *N* = 885	Short (<22)	39	+0.043	−0.003	+0.115	+0.085	0.429	0.411	0.429	0.421
	Normal (22–25)	637	−0.008	−0.003	+0.008	+0.000	0.376	0.357	0.359	0.358
	Long (>25)	209	+0.042	+0.015	−0.049	−0.037	0.329	0.305	0.309	0.307
ZCB00 *N* = 612	Short (<22)	76	+0.050	+0.031	+0.110	+0.011	0.535	0.478	0.486	0.477
	Normal (22–25)	467	−0.010	−0.005	−0.012	−0.001	0.388	0.367	0.366	0.367
	Long (>25)	69	+0.065	+0.026	−0.051	+0.043	0.381	0.350	0.349	0.352

**Table A3:** Subgroup analysis by Anterior Chamber Depth. Mean prediction error (ME) and RMS PE stratified by phakic anterior chamber depth (ACD). Shallow: ACD < 2.8 mm; Normal: 2.8 ≤ ACD ≤ 3.4 mm; Deep: ACD > 3.4 mm. Formulas A–D as in Table A1.

IOL (*N*)	ACD Subgroup (mm)	*n*	ME A (D)	ME B (D)	ME C (D)	ME D (D)	RMS A (D)	RMS B (D)	RMS C (D)	RMS D (D)
Clareon *N* = 142	Shallow (<2.8)	26	+0.045	−0.016	+0.047	+0.011	0.319	0.309	0.306	0.306
	Normal (2.8–3.4)	74	−0.021	−0.018	−0.018	−0.024	0.344	0.336	0.341	0.337
	Deep (>3.4)	42	+0.027	+0.041	+0.007	+0.018	0.410	0.398	0.407	0.400
MX60 *N* = 467	Shallow (<2.8)	59	+0.089	+0.047	+0.125	+0.086	0.381	0.366	0.395	0.381
	Normal (2.8–3.4)	260	−0.006	−0.029	−0.011	−0.038	0.382	0.356	0.361	0.360
	Deep (>3.4)	148	+0.022	−0.026	−0.039	−0.059	0.400	0.398	0.415	0.412
SA60AT *N* = 821	Shallow (<2.8)	228	+0.008	−0.008	−0.023	+0.017	0.454	0.435	0.440	0.436
	Normal (2.8–3.4)	432	−0.005	+0.001	+0.000	−0.016	0.390	0.377	0.378	0.380
	Deep (>3.4)	161	+0.027	+0.015	+0.031	−0.026	0.426	0.406	0.411	0.409
SN60WF *N* = 254	Shallow (<2.8)	43	+0.009	−0.004	+0.111	+0.098	0.332	0.327	0.364	0.364
	Normal (2.8–3.4)	128	−0.015	−0.028	−0.010	−0.034	0.376	0.366	0.375	0.380
	Deep (>3.4)	83	+0.039	+0.007	−0.049	−0.080	0.388	0.376	0.381	0.388
Vivinex *N* = 885	Shallow (<2.8)	148	+0.029	−0.022	+0.018	+0.003	0.400	0.373	0.378	0.376
	Normal (2.8–3.4)	462	−0.025	−0.030	−0.027	−0.033	0.373	0.352	0.354	0.354
	Deep (>3.4)	275	+0.047	+0.067	+0.034	+0.037	0.341	0.327	0.331	0.328
ZCB00 *N* = 612	Shallow (<2.8)	109	+0.016	−0.002	+0.027	−0.008	0.457	0.429	0.434	0.427
	Normal (2.8–3.4)	335	−0.010	−0.007	−0.008	−0.006	0.404	0.379	0.380	0.380
	Deep (>3.4)	168	+0.030	+0.027	−0.007	+0.035	0.383	0.349	0.344	0.351
